# Advancements in cancer biomarker discovery over fifteen years: diagnostics and prognostic approach in somatic cancer

**DOI:** 10.3389/or.2026.1809836

**Published:** 2026-05-08

**Authors:** Mohd Basheeruddin, Sana Qausain, Ashish Anjankar, Faez Iqbal Khan

**Affiliations:** 1 Department of Biochemistry, Jawaharlal Nehru Medical College, Datta Meghe Institute of Higher Education and Research, Wardha, India; 2 Department of Biosciences and Bioinformatics, School of Science, Xi’an Jiaotong-Liverpool University, Suzhou, China

**Keywords:** biomarkers, cancer, genomics, liquid biopsy, personalized medicine, proteomics

## Abstract

Cancer biomarkers are now recognized as major tools for early detection, diagnosis, and prognosis. In the last 10 years or so, molecular biology, proteomics, and genomics have been rapidly advancing to find tissue-specific biomarkers used in clinical practice. In this review, the development of the cancer biomarkers published in 2011–2025, for lung, breast, liver, and kidney cancers, is reviewed. A systematic review of peer-reviewed literature searched PubMed, Scopus, and Web of Science, including human and published English studies. A variety of detection approaches, including immunohistochemistry, next-generation sequencing, and liquid biopsy technology, were assessed with an explicit focus on clinically relevant biomarkers. The main trends indicate that while classic protein markers, particularly carcinoembryonic antigen and neuron-specific enolase in lung cancer, hormone receptor status and HER2 in breast cancer, and alpha-fetoprotein in liver cancer, have evolved, many modern genomic markers including epidermal growth factor receptor mutations, anaplastic lymphoma kinase rearrangements, TP53 mutations, vascular endothelial growth factor pathways, and von Hippel–Lindau gene alterations have evolved, resulting in a large gap in their knowledge. These innovations underscore the importance of molecular biomarkers in supporting early detection, targeted therapy, and enhanced surveillance of disease progression. Cancer biomarker studies have evolved from protein-based biomarkers to encompass both genomic and transcriptomic targets, which may allow for more targeted and individualized cancer interventions. Multi-omics integration and novel types of biomarkers like circulating tumor DNA, circulating tumor cells, and microRNAs will focus on developing early diagnosis, prognosis, and personalized treatment strategies as a prerequisite of such work.

## 1 Introduction

Cancer biomarkers form the nucleus of modern oncology as they are necessary for the diagnosis, prognosis estimation, and therapeutic guidance. Development of molecular biology and genomic technology has widened the range of biomarkers discovered in multiple cancers and enhanced diagnosis accuracy and the prospect for precision medicine (or “medicine”) in clinical oncology (1).

### 1.1 Background of the topic

A biomarker is a measurable indicator of a biological state that reflects typical physiological processes, development of disease, or response to treatment. In the domain of oncology, biomarkers are commonly expressed in tissues, blood or other biological fluids and have been identified to provide important information on the existence and development of cancer (2). Cancer biomarkers are typically divided into diagnostic, prognostic, and predictive. Diagnostic biomarkers confirm the carcinogenic agent existent; prognostic biomarkers are indication of disease outcome and progression; predictive biomarkers demonstrate the potential outcome of a given therapeutic intervention (3).

The past decade has seen the advancement of molecular biology and high-throughput technologies to greatly enhance cancer biomarker discovery. Genomic and molecular analysis based techniques enable the recognition of molecular phenotypes describing an individual’s tendency towards cancer and therapeutic response (4). Given the different biological and genomic characteristics of tumors, there is an increasing need for tissue-specific biomarkers with molecular representations of these pathological changes in diverse cancers, such as lung, breast, liver and renal malignancy (5). These biomarkers can also help in diagnosis as well as in planning of treatment and monitoring for therapeutic response and detection for disease recurrence (6).

### 1.2 Importance of the research area

Cancer continues to exert a substantial global health influence and timely diagnosis and targeting of treatment pathways are critical to enhancing patient outcomes. This made biomarkers a significant part of oncology because they offer valuable information about tumor biology and guide clinicians in making therapeutic judgments. Applying new genomic technologies like next-generation sequencing (NGS) has facilitated the thorough characterization of genetic alterations linked with development and progression of cancer (7). From these technological advancements, clinicians can identify molecular targets and start precision-based therapeutic methodologies.

Over the past years, the regulatory approvals of translatable technologies (e.g., liquid biopsy and circulating tumor DNA (ctDNA) analyzing) have brought forth new opportunities in non-invasive cancer monitoring and disease management (8). These progressions notwithstanding, there are hurdles to the use of biomarkers in routine clinical practice. Differences in tumor biology, low sensitivity and specificity of some biomarkers, no standardized testing procedure, and financial and infrastructure constraints limit their general use in the clinical setting. In addition, new technologies like artificial intelligence take a lot of resources and regulations to ensure it’s integrated into routine practice. As a result, ongoing research and validation are required to guarantee the robust use of biomarkers in the clinic.

### 1.3 Aim or objective of the review (narrative review)

In this narrative review, key biomarkers relevant for routine cancers with a focus on lung, liver, breast, and renal malignancies are reviewed. The paper considers clinical, molecular and biochemistry value of established and emerging biomarkers, and summarizes recent technological developments that have contributed to their identification and usage. These can include the biomarkers EGFR mutations, ALK rearrangements, and PD-L1 expression of lung cancer, GP73 and ctDNA of liver cancer and mutations of BRCA and gene-expression panels like Oncotype DX in breast cancer (9, 10). For renal cancer, biomarkers such as VEGF, VHL mutations and PD-L1 expression are crucial to inform targeted therapy and immunotherapy see Table 1 (11).

**TABLE 1:** Comprehensive table summarizing key cancer biomarkers and detection advancements from the past 15 years.

Cancer and years	Aims and objectives	Advantages	Limitations	Remarks	References
Lung cancer (2009–2024)	EGFR, ALK, PD-L1 biomarker targeting	Improves patient selection and survival	Drug resistance; limited responders	Key advance in precision therapy for NSCLC	(12)
Liver cancer (2009–2024)	AFP, GP73, ctDNA for diagnosis	Non-invasive early detection and monitoring	Variable sensitivity; elevated in benign liver disease	Early detection still challenging	(13)
Breast cancer (2009–2024)	Oncotype DX, BRCA, HER2 testing	Guides therapy; predicts recurrence	High cost; limited accessibility	Improves treatment decisions	(14)
Renal cancer (2009–2024)	VEGF, VHL mutation, PD-L1 targeting	Enables targeted therapies	VEGF inhibitor resistance; immune evasion	Requires further clinical validation	(15)

This review aims to elucidate the emerging role of biomarker technologies in cancer diagnosis and treatment by focusing on ongoing developments as well as the current hurdles such as therapeutic resistance, validation challenges, and accessibility problems. This aims to facilitate the professional development of clinicians and researchers for precision oncology and patient-centered cancer treatment.

## 2 Methodology

### 2.1 Literature search strategy

This study was performed as a narrative review with the goal of collating recent status and new opportunities for cancer biomarker studies published from 2011 to 2025. A structured literature search was carried out in order to identify pertinent peer-reviewed studies on the identification of biomarkers, diagnostic applications, prognostic importance, therapeutic management in oncology. A search was performed through the leading biomedical and multidisciplinary databases, in this case PubMed, Scopus and Web of Science, which included a wide-ranging review of scientific publications in the fields of biomedical sciences, molecular biology and clinical oncology. Beyond the searches on the database, literature reviews used manual screening of reference lists in literature from chosen review articles and articles in order to include all relevant studies. Search queries were performed by searching with combinations of keywords about cancer biomarker research, such as “cancer biomarkers,” “tumor biomarkers,” “molecular biomarkers,” “liquid biopsy,” “precision oncology,” and “next-generation sequencing.” Boolean operators (AND + OR) were used to refine the search and improve the reliability of the results obtained. A representative search strategy included: (“cancer biomarkers” OR “tumor biomarkers”) AND (“genomics” OR “proteomics”) AND (“diagnosis” OR “prognosis” OR “therapy”).

The search was limited to work from January 2011 to March 2025 to cover a relatively recent and extensive analysis of biomarker applications and technology. To ensure comparability in analysis findings, only English language articles were included. Retrieved articles were screened for their title and abstract relevance to the study topic, and those that were considered relevant were fully assessed through full text analysis. Given the primary aim of this work was to provide a conceptual and thematic synthesis of current knowledge, the study was conducted as a narrative review; it was not done systematically.

### 2.2 Inclusion criteria

For this review, studies were selected if they met eligible criteria relevant to the objectives of the study. Eligibility criteria were that articles were published within peer-reviewed scientific journals related to the discovery, validation or clinical applications of cancer biomarkers. Among the articles included, studies related to human clinical trials or translational investigations with demonstrable relevance to the domain of human oncology provided the most robust evidence for clinical utility. Furthermore, studies in molecular biomarkers for genomics, proteomics, transcriptomics, and liquid biopsy technologies were also included, as these fields are also important technological developments toward modern cancer diagnosis and precision medicine. Articles published in English between 2011 and 2025 and used to cover news of recent scientific advancements. We included original research articles alongside review articles, to illustrate the existing knowledge and emerging trends in cancer biomarker studies.

### 2.3 Exclusion criteria

Studies excluded from the review were the ones that did not meet this inclusion or exclusion criteria. Conference abstracts, editorials, commentaries and other non-peer-reviewed material, were not included as most of those may not have available methodological details and comprehensive data. To preserve scientific reliability in the literature selected, articles without sufficient experimental or methodological detail were also excluded. Studies focusing exclusively on animal models without direct translational relevance to human cancer—the focus of this review—were excluded because their work was on biomarkers of biological significance for human clinical research and precision oncology. Papers that were not specifically relevant in cancer biomarker discovery, diagnostic applications, prognostic evaluation, or therapeutic monitoring were also excluded. We used these inclusion and exclusion criteria to analyze the selected studies in a systematic way, categorizing the included studies by biomarker category, technology, and clinical applications in the field of oncology, allowing for a systematic synthesis of available evidence.

## 3 Biomarkers in major cancer types

### 3.1 Lung cancer biomarkers

Lung cancer is still a crucial public health issue on an international scale. Non-small cell lung cancer (NSCLC) is responsible for the largest mortality rates due to cancer globally. By leveraging molecular oncology, we have now identified key biomarkers involved in the diagnosis, prognosis, and treatment of lung cancer (16). These include Epidermal Growth Factor Receptor (EGFR), KRAS (Kirsten Rat Sarcoma Viral Oncogene Homolog) mutations and Programmed Death-Ligand 1 (PD-L1), which have become standard biomarkers for the development of targeted therapy regimens in the modern medical treatment landscape (17). Mutational biomarkers such as EGFR, ALK, and ROS1 are employed for testing the compatibility of targeted therapy and the therapeutic outcomes improving patients benefits. Biomarkers for prediction of future evolution of the disease and overall survival rates, such as TP53 mutations, circulating tumor DNA (ctDNA) and tumor mutational burden (TMB) (6), are also used similarly. To enhance early identification and personalized therapy of lung cancer, the determination and further confirmation of these biomarkers is mandatory (18). NGS can detect genetic alterations which influence the pathways that predispose to lung cancer such as EGFR, KRAS and ALK. These systems enable the provision of appropriate care, outcomes prediction, and resistance diagnosis to therapy, thus increasing the specificity treatment outcomes in lung cancer (19).

#### 3.1.1 Epidermal growth factor receptor (EGFR)

Receptor tyrosine proteins found on the cell membrane are important for regulating growth and survival. Epidermal Growth Factor Receptor (EGFR) is one of the most familiar receptors, and is mainly responsible for signaling pathways related to cell proliferation, differentiation and survival. Mutations of the EGFR gene are common in Non-Small Cell Lung Cancer (NSCLC), especially in lung adenocarcinomas, and in patients with a less or no history of smoking. These mutations lead to abnormal activation of downstream signaling pathways, resulting in uncontrolled cell growth and tumor progression (20). Such mutations are good for targeting by tyrosine kinase inhibitors (*TKIs*) like erlotinib, gefitinib, and afatinib that work by inhibiting *EGFR* signaling and thus, slowing down tumor progression. These molecular therapies have brought about tremendous progress of PFS of the patients with mutants of *EGFR* (21). However, development of resistance to *TKIs* is common and the second mutation that occurs is T790M. To this end, new generation of *TKIs* including osimertinib have been designed to overcome the resistance and hence increase survival (22). The ability to hit EGFR mutations brings out the efficiency of precision medicine in the treatment of the lung cancer. Thus, the emergence of resistance should continue the efforts on the search for new drugs at the same time, consider the possibility of using combined therapy to obtain more favorable long-term outcomes (23).

#### 3.1.2 KRAS (Kirsten rat sarcoma viral oncogene homolog) mutations

*KRAS* is one of the most common oncogenes affected in NSCLC, with mutations identified in 20%–30% of adenocarcinomas of smokers. They induce uncontrolled cell proliferation and are usually associated with malignant tumor characteristics (24). Indeed, KRAS mutations are generally linked with poor prognosis and, in particular, *TKIs,* although their treatment has been historically considered difficult. But thanks to recent progress, *KRAS G12C* inhibitors like sotorasib have been designed to target only the mutated *KRAS* proteins. This new therapy has brought light to patients with little or no other treatment options (25). That *KRAS* mutations are known to be such effective targets clearly points to the future of smart drug design and possible design of drugs against some of the other previously known ‘undruggable’ targets (26).

Certain *KRAS* gene polymorphisms have been linked in studies to the onset and progression of lung cancer. Specifically, let-7 microRNAs cannot bind to *KRAS* due to the rs712 and rs61764370 variations in the 3′untranslated region (3′UTR) of *KRAS*. This increases the production of *KRAS*, which raises the risk of cancer, mainly in women and nonsmokers (27). Additionally, the intronic mutation rs12587 has been linked to changes in *KRAS* regulation and may contribute to the development of lung cancer. Somatic mutations *G12D, G12V*, and *G12C* are important in conjunction with SNPs because they generate oncogenic alterations and raise the likelihood of poor outcomes and treatment resistance in patients with non-small cell lung cancer (NSCLC) (28).

*GTPase,* one of its main functions which is encoded by the *KRAS* gene and is an essential protein in the RAS/MAPK pathway responsible for overseeing cell growth, differentiation and protection. When everything works reasonably well, *KRAS* switches between *GTP* and *GDP* forms to send information from *EGFR* to the nucleus. When *KRAS* codons 12, 13 or 61 mutate in cancer, it remains stuck in the active GTP-bound form. Consequently, cellular functions are controlled incorrectly and cells multiply continuously, apoptosis is prevented and the tumor rapidly progresses (29).

Changes in KRAS genes are present in many human cancers, especially non-small cell lung cancer (NSCLC), colorectal cancer and pancreatic cancer. They are strongly associated with an unfavorable prognosis, fast-advancing cancers and unresponsiveness to EGFR inhibitors. KRAS functional and mutation mechanisms contribute to the selection of an appropriate treatment modality and the prediction of response (30).

#### 3.1.3 Programmed Death-Ligand 1 (PD-L1)

**FIGURE 1:**
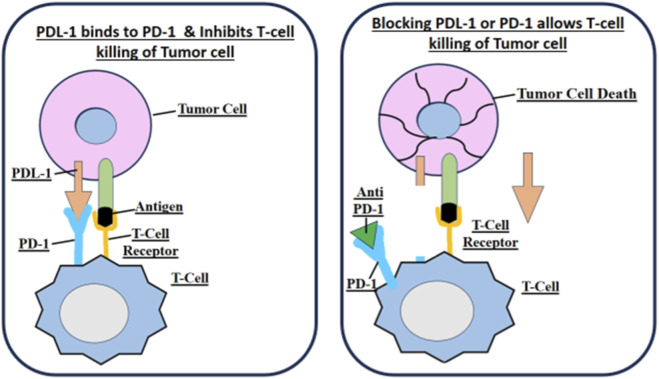
PD-L1/PD-1 Blockade- how immune checkpoint inhibitors restore T-cell function by disrupting the PD-L1/PD-1 interaction.

Programmed Death-Ligand 1 (PD-L1) is a protein on the surface of tumor cells that suppresses T-cell stimulation so as to escape immune recognition by tumors. Expression of PD-L1 is an important biomarker to guide immunological decisions for NSCLC treatment (31). Immune checkpoint inhibitors such as pembrolizumab, nivolumab and atezolizumab inhibit the interaction between PD-L1 and its receptor PD-1 as illustrated in Figure 1, thus regaining immune surveillance and allowing the immune system to attack cancer cells (32).

PD-L1 expression levels, clinically, to settle patients for treatment eligibility Patients with NSCLC showing high PD-L1 expression of 50% or more may benefit extensively from immunotherapy as a first-line therapy, while those with lower expression levels may need combination therapy, including chemotherapy or targeted drugs for therapy (33). PD-L1-guided immunotherapy is now central to precision oncology and delivers a reliable responsive approach in high biomarker-expressing patients. Meanwhile immunization-supported immune checkpoint inhibitors coupled with alternative medications continue to broaden treatment choices to many individuals (34).

### 3.2 Breast cancer biomarkers

Global breast cancer is still one of the most growing cancers to cause the concern for the global health squad. This advancement toward molecular oncology, has established robust biomarkers that can be challenging to diagnose, estimate, and treat breast cancer. These include HER2, ER, and PR which have become vital components of the tailored treatment programs (35).

NGS is key to finding breast cancer biomarkers through looking for mutations in TP53, PIK3CA, BRCA1/2. Thanks to NGS we have developed a detailed understanding of the tumors for diagnosis, accurate forecasting, and disease specific selection of therapeutic approaches (19). HER2 amplification, ER positivity, and BRCA 1/2 mutations are some of the types of predictive biomarkers targeted for these patients who are receiving trastuzumab for HER2 positive cancers and endocrine treatment for hormone receptor positive breast cancer (36). Likewise, molecular prognostic markers such as ki-67, TILS score and CTCs give useful information about disease aggressiveness, recurrence rates, and survival rates. These biomarkers have to be identified and affirmed continuously to enhance the early diagnosis and the use of precision medicine, and improve the treatment of breast cancer (37).

#### 3.2.1 Human epidermal growth factor receptor 2 (HER2)

*HER2* is a transmembrane glycoprotein which is also a receptor of the epidermal growth factor that is involved in cell growth and survival, and may also induce differentiation. The *HER2* receptor is overexpressed or amplified in about 15–20 percent of breast cancer cases and is linked with high tumour aggressiveness and unfavorable prognosis (38). *HER2* status is an essential biomarker directing therapies including trastuzumab, pertuzumab and *T-DM1*, that interferes with *HER2* signaling and prevents cancer cell proliferation. Both of these treatments have boosted survival rates of patients diagnosed with *HER2*-positive breast cancer. Nevertheless, there has been increasing concern with regard to the resistance to *HER2* targeted therapies often because of changes to downstream signaling pathways or expression of receptors. New combinations of the drugs and the development of new targeted *HER2* agents for *HER2* positive-breast cancer continue to grow with the goals of overcoming resistance and improving patients’ experiences (39).

#### 3.2.2 Estrogen receptor (ER)

Estrogen Receptor or ER is a nuclear intracellular receptor that helps in the regulation of gene expression through activities of estrogen. ER is the most common biomarker for the subtype classification of breast cancer, with 70%–80% of breast cancers being ER-positive. ER-positive breast cancers are typically more favorable and sensitive to endocrine therapy by drugs that can block the action of estrogen that promotes cell division leading to tumor (40). Nevertheless, all these therapies are problematic in that patients can develop resistance to them, it primarily resulting from genetic mutations, such as those in the ESR1 gene, or the activation of other signaling networks. Work in the combination of endocrine therapies with CDK4/6 inhibitors or PI3K inhibitors has been found to help in overcoming resistance and hence increase progression-free survival. ER still remains relevant as a diagnostic, prognostic and predictive biomarker throughout the management of HR positive breast cancer (41).

#### 3.2.3 Progesterone receptor (PR)

The Progesterone Receptor (PR) is another nuclear hormone receptor that, like ER, is activated by hormones to regulate gene transcription. PR expression is often co-expressed with ER in breast cancers and serves as a marker of functional estrogen signaling. PR positivity is generally associated with a favorable prognosis and better response to endocrine therapy (42). However, its role as a standalone biomarker is limited, as PR status is primarily used in conjunction with ER and HER2 to classify breast cancer subtypes and guide treatment decisions. Studies suggest that the loss of PR expression in ER-positive cancers may indicate a more aggressive phenotype and a higher likelihood of endocrine resistance. Future research into the molecular mechanisms governing PR signaling and its interaction with ER may help refine its clinical utility and guide personalized treatment strategies (43).

#### 3.2.4 Recently detected biomarkers for breast cancer risk and progression

Recent technological advancements have allowed researchers to discover a large number of novel biomarkers linked to conditions including the onset and spread of breast cancer. *PIK3CA, TP53*, and *GATA3* mutations are frequently observed in breast cancers and are linked to the tumor’s development, metastasis, and therapy insensitivity. One of the main indicators of hereditary breast cancer is the presence of mutations in the *BRCA1* and *BRCA2* genes. CtDNA and microRNAs (such as miR-21 and miR-155) are gradually being investigated to find potential use in non-invasive cancer monitoring and detection (44).

### 3.3 Liver cancer biomarkers

Liver cancer, predominantly HCC, is still one of the most lethal cancers, its incidences being on the rise all around the world. New discoveries in molecular oncology have helped in the discovery of biomarkers that play a important role in the diagnostic, prognostic and therapeutic considerations of liver cancer. Of those, AFP, Hepatitis B Virus (HBV) DNA, and c-MET appear to be the most relevant biomarkers for designing customized therapeutic approaches (45).

Liver cancer biomarkers are often found through NGS which detects changes in genes such as TP53, *CTNNB1* and *AXIN1*. Because of NGS, comprehensive genomics can be done, leading to better treatment planning and outcomes for patients with liver cancer (46).

Serum AFP, HBV and HCV markers, genetic alterations like TP53 or c - MET aberrations determines targeted therapies like TK inhibitors, sorafenib in advanced HCC (47). Diagnostic biomarkers and prognostic biomarkers encompass AFP, miRNAs and ctDNA as they establish information about disease progression, recurrence and survival rate. Further identification and confirmation of such biomarkers are crucial for enhancing the diagnostic capability in the early stage, refining the concept of personalized satisfactory treatment for liver cancer (48).

#### 3.3.1 Alpha-fetoprotein (AFP)

**FIGURE 2:**
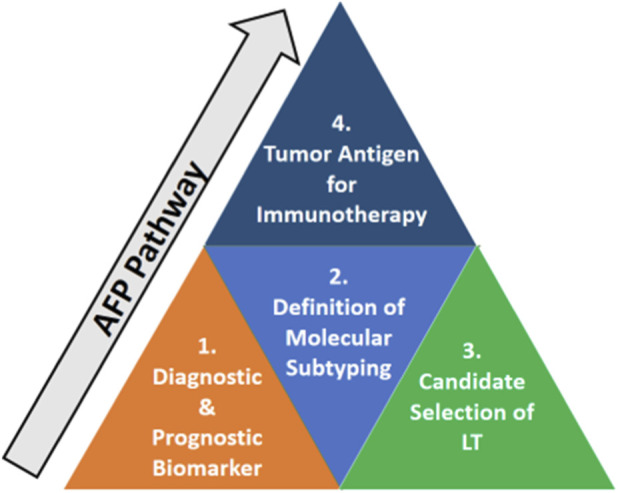
Clinical roles of alpha-fetoprotein (AFP) in liver disease and hepatocellular carcinoma.

It is a glycoprotein mainly synthesised by the fetal liver, yolk sac and gastrointestinal tract and is used as a tumour marker. In adults, the increased level of AFP is linked to liver illnesses, and more specifically, HCC and other types of cancer too. AFP is valuable for the diagnosis of HCC and the assessment of the disease progression, since AFP concentration is higher in individuals with this cancer (49). Even though AFP can indicate the presence of liver cancer, its performance indicators are not highly effective; it is utilised jointly with imaging procedures such as ultrasound or CT. Further, AFP levels as shown in Figure 2, can be measured during treatment to evaluate the response to treatment or reappearance of cancer (50).

Nevertheless, AFP still holds its usefulness in dealing with liver cancer, particularly those patients with high risk factors or determinant conditions such as chronic hepatitis B or C, cirrhosis or family history of liver cancer (51).

#### 3.3.2 Hepatitis B virus (HBV) DNA

Serum Hepatitis B Virus (HBV) DNA is an effective tool in diagnosis and management of chronic Hepatitis B Virus infections that causes liver diseases like cirrhosis and HCC. The detectable forms of HBV in circulation are HBeAg and HBV DNA, both of which reflect active viral replication, the main pathogenic mechanism in liver injury and carcinogenesis (52). HBV DNA quantitation is crucial in determining viral replication, prognosis of the disease process and in management of antiviral treatment. Current high levels of HBV DNA are also associated with an increased risk of liver cancer and antiviral therapy seems to be effective in reducing the risk (53). Moreover, measurement of HBV DNA is necessary for assessment of therapeutic outcomes and identification of cases of treatment failure, to ensure that proper changes in the therapeutic plan are made. Because of its significance in the larger approaches towards liver cancer prevention and care, HBV DNA testing cannot be overlooked in the current management of at-risk patients (54).

#### 3.3.3 c-MET

c-MET is also known as mesenchymal-epithelial transition factor or metastasis associated protein and is a receptor tyrosine kinase involved in cell survival, proliferation, migration and angiogenesis (55). In hepatocellular carcinoma (HCC), c-MET is overexpressed or mutated and have prognostic effect for tumor progression, metastasis and chemoresistance Conversely, c-MET signaling was found to be stimulated through its ligand, Hepatocyte grow factor (HGF), Hence this pathway has involvement in pathway such as epithelial-mesenchymal transition (EMT),which enhance cancer cell invasiveness (56). These two articles suggest that from ordinary perspective, c-MET has been activated abnormally in HCC, which results in poor prognosis and resistance to treatments such as sorafenib. Thus, c-MET was identified as target for molecular based therapy in HCC. Another type of targeted therapies under development includes c-MET inhibitors which are still in trial to overcome resistance to enhance patient survival (57). Moreover, c-MET is also usable as a prognostic and predictive marker since the higher expression of c-MET correlates with more aggressive phenotype and increased tendency to metastasize (58).

### 3.4 Renal cancer biomarkers

RCC is one of the most prevalent malignant tumours of the kidney, posing a major threat to human life due to cancer. New discoveries made in molecular oncology have identified significant biomolecules that play a central role in diagnosis, assessing tendencies, and management of renal cancer (59). Molecular markers including *VEGF, EGFR*, and *CD31* are pivotal in the management of targeting therapy in RCC. These markers are significant in the determination of treatment plans which can zero to knockout molecular targets that cause cancer growth and spread. As for genetic changes involved in RCC development, VHL (von Hippel-Lindau) gene is particularly important because its mutations play a major role in RCC and provide information on the disease’s development that can be useful in treatment (60).

Beyond the predictive biomarkers, the prognostic biomarkers including Ki-67 status, TILs, and ctDNA are critical moving forward and estimating the risk of recurrence. High Ki-67 labeling index is represented in higher tumor grade and increased cell proliferation rate therefore a poor prognosis (61). The findings of TILs and ctDNA give additional information regarding the tumor progression and probability of metastatic disease. It is quite essential to identify and validate these biomarkers to enhance effective intervention during the availability of personalized treatment (62). Continued identification and incorporation of new biomarkers into practice will not only improve the detection of renal cancer at an early stage, but also allow the targeted therapy to be applied to the tumor (63). Renal cancer biomarkers are advanced by NGS, since it detects mutations in popular genes such as *VHL, PBRM1* and *SETD2*. The detailed review of genetics provides more accurate diagnosis, risk categorization and therapy decisions for people with renal cell carcinoma which enhances their treatment results and the chances of a good prognosis (64).

#### 3.4.1 Vascular endothelial growth factor (VEGF)

Vascular Endothelial Growth Factor (VEGF) is also a signaling receptor involved in formation of new blood vessels a process called Angiogenesis. One example is that *VEGF* is overexpressed in renal cell carcinoma (RCC) and serves to help tumor growth by creating new blood vessels that feed the tumor (65). *VEGF* plays a role as a molecular factor targeted for the therapy that works to prevent tumor angiogenesis. Multi-targeted tyrosine kinase inhibitors (*TKI*) like sunitinib and pazopanib act by inhibiting *VEGF* which in turn, inhibit angiogenesis and tumor growth (65). To a greater extent, these targeted therapies are effective in RCC patients based on their ability to enhance progression-free survival. But there might be resistance to such therapies, and thus further research needs to be conducted to find new reagents and combinations of *VEGF*-targeting drugs in order to avoid or reverse possible resistance mechanisms and thus better patient outcomes (65).

#### 3.4.2 Epidermal growth factor receptor (*EGFR*)

Epidermal Growth Factor Receptor also known as *HER1, ErbB-1* or member of the *ErbB* family of receptor tyrosine kinases is a cell surface receptor involved in cell growth and differentiation. The presence of *EGFR* is associated with RCC and its overexpression results to stimulus of cell proliferation (66). Molecular targeted therapies involving *EGFR* signaling pathway in RCC are under consideration; the drugs being gefitinib and erlotinib. These therapies are designed to inhibit *EGFR* to decrease cell growth in tumor and its expression (67). However, as in other targeted therapies, resistance to *EGFR* inhibitors may be encountered, thus further studies to find second generation *EGFR* inhibitors or combined therapies, which will provide better treatment outcomes persist (68).

#### 3.4.3 Cluster of differentiation 31 (CD31)

**FIGURE 3:**
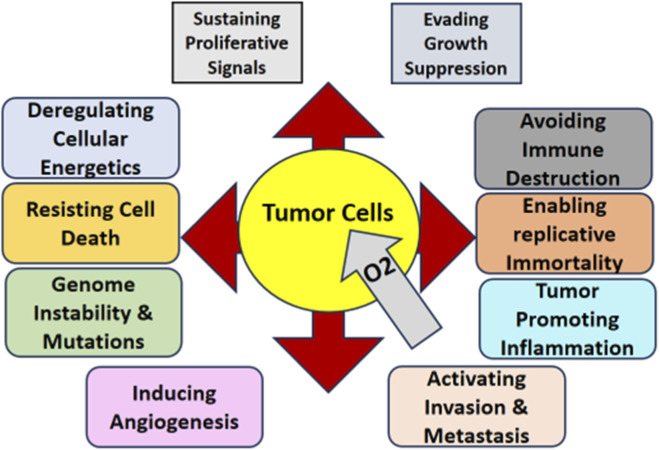
Hallmarks of cancer associated with tumor progression (Hanahan D, Weinberg RA. Hallmarks of cancer: the next-generation. Cell. 2011; 144 (5):646–674.).

*CD31,* also called as *PECAM-1,* is a member of the immunoglobulin superfamily and is found on platelets, endothelial cells and certain leukocyte populations and plays an essential role in endothelial cell signaling, mediation of angiogenesis. CD31 antigen is well recognized in endothelial cells of blood vessels and it has an important function in the stability of vascular network (69). *CD31* expression has been used in the context of RCC to predict the tumor microvascular density and the aggressiveness of the tumor. Targeting *CD31* in RCC offers a new strategy to since cell surface molecules are important regulators of cell functions, they can be used to stop the tumor growth and development (70). Since *CD31* may interact with endothelial cells and inhibit angiogenesis, these therapies may provide better treatment options that are more effective than currently available *VEGF* inhibitors in RCC (71), and new treatment options for patients with advanced RCC are shown in Figure 3.

Genetic and epigenetic molecular biomarkers are now an integral part of cancer diagnosis and management because they afford information about cancer cells, their behaviour and response to treatment. Common molecular alterations include *TP53* that is mutated in fifty percent of all cancers and is portending a worse survival rate (72). Amongst recommended biomarkers, *TP53*, better known as the “guardian of the genome”, is involved in cell cycle arrest, DNA repair and apoptosis. They cause the loss of the tumor-suppressing activity and so lead to uncontrolled cell proliferation and tumor development (73). It not only corresponds to the severity of the disease but also acts as potential therapeutic markers of cancers such as lung, and breast cancer. Due to this it is possible for clinicians to determine particular gene changes and therefore the survival likelihood of the patient and this improves the efficiency of the available cancer remedies (4).

NGS is important for finding molecular biomarkers by looking simultaneously at DNA sequencing and chemical changes on the DNA. The test finds differences in DNA, changes in its number of copies and marks on DNA and histones called methylation and modifications. Learning about both genetic and epigenetic causes contributes to better diagnosis and prognosis which helps select targeted therapy methods (7).

DNA methylation and histone modification are also important components in the development of cancer biomarkers. Breast cancers, for example, are associated with deviant levels of DNA methylation for instance, high levels of DNA methylation for specific genes, the promoters of most tumor suppressor genes (74). Hyper methylation also is also a mechanism of silencing tumor suppressor genes which causes interference with normal cellular regulation thus facilitating malignant transformation. Likewise, post translational modifications of histones such as acetylation and methylation are implicated in chromatin remodeling and gene expression regulation. Epigenetic alterations are particularly prominent in cancers such as leukemia and lymphoma, where they play a critical role in the activation or repression of gene expression. These epigenetic changes add not only diagnostic and prognostic significance but also potential targets for treatment since epigenetic modifications are dynamic (75). Promising examples include the continuing development of epigenetic therapy, such as drugs that target DNA methyltransferases and histone deacetylases, to show the direction in which these biomarkers can be used in cancer treatment. Genetically and epigenetically derived biomarkers fall into a unified platform in deciphering tumor heterogeneity, decision making in precision medicine and enhancing the quality of survival in cancer related ailments (76).

## 4 Emerging biomarkers and technologies

**FIGURE 4:**
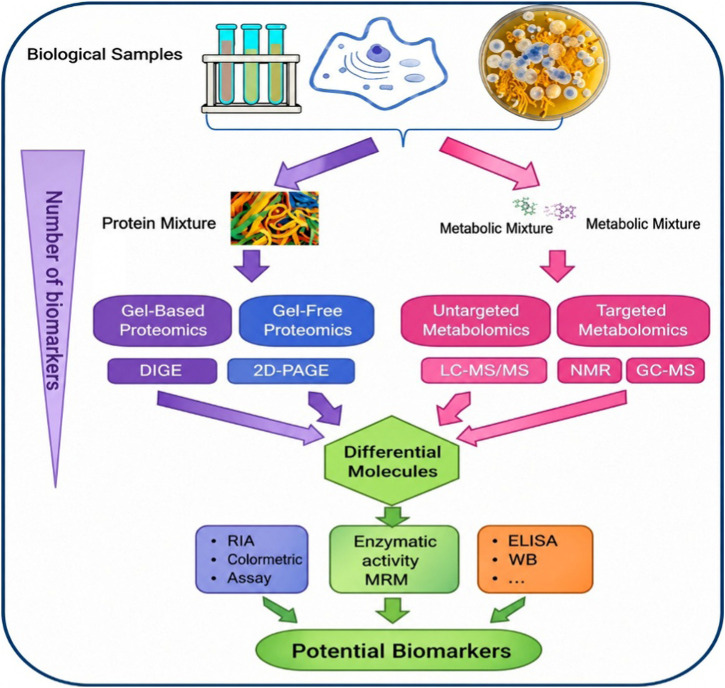
Integrated workflow for biomarker discovery using proteomics and metabolomics technologies.

Biological samples are analyzed using advanced proteomic and metabolomic approaches to identify novel biomarkers. Protein mixtures are studied through gel-based and gel-free proteomics techniques such as DIGE and 2D-PAGE, while metabolic mixtures are evaluated using LC-MS/MS, NMR, and GC-MS is depicted in Figure 4. Differential molecules identified are further validated through enzymatic assays, RIA, ELISA, and Western blot to establish potential clinical biomarkers.

### 4.1 Liquid biopsy

Liquid biopsy is a non-invasive diagnostic and monitoring approach for cancer that involves the detection and analysis of tumor-derived biomarkers present in body fluids such as blood and other biological fluids. *CtDNA* is dynamic and used to monitor tumor evolution in real-time and identify resistance mechanisms to build a personalized approach to treatment (8). Exosomes are secreted cells that possess characteristics of cancers and transmit cancer-associated proteins and nucleic acids. They have great potential for not only being diagnostic and prognostic factors underlining disease development but also yielding information about treatment outcomes. Liquid biopsy has become a promising addition to the conventional solid tissue biopsy, and helped to increase the accuracy of cancer interventions (77).

### 4.2 Non-blood biofluids

Non-blood fluids such as saliva, urine, stool and sweat offer possibilities for painless and easy ways to diagnose and screen cancers, particulary in children and elderly people. Saliva sample analysis discovers residue of DNA, RNA and different proteins that can detect cancers such as those in the mouth and lungs. Many types of tumor-specific biomarkers are found in urine, so it can assist with the detection of urological and other cancers. Stool is currently used as a method of screening for colorectal cancer by using DNA and immunochemical tests (78). Though sweat is also being studied, it might expose metabolic or proteomic indicators for cancer. This means people can easily take samples themselves and get diagnosed quickly, building better habits and leading to better personalized cancer care. Having medication administered routinely will continue to require clinical validation and standardization (79).

### 4.3 Immunotherapy biomarkers

The discovery of immunotherapies, which appears to have a biomarker for treatment outcomes, is instructive. Tumor Mutational Burden (TMB) as remained one of the essential biomarkers that associate with improved efficacy of immunotherapy across immune checkpoint inhibitors and heightened tumor immunogenicity. Furthermore, neoantigens that are tumor-specific antigens due to a genetic mutation engaged in activating the immune response (80). These tumor-associated antigens are currently being investigated for the development of patient-specific cancer vaccines and are expanding the field of cancer immunotherapy. Furthermore, by identifying patients who are more likely to benefit from immunotherapy, these biomarkers significantly enhance the precision and effectiveness of immunotherapeutic strategies in the treatment of malignant diseases (81).

### 4.4 Long non-coding RNAs (lncRNAs) and miRNA in cancer development and progression

Experts now concur that lncRNAs promote the development and spread of breast cancer. Protein-coding genes only affect gene expression on a single level, however lncRNAs like HOTAIR, MALAT1, and BCAR4 affect gene expression in several ways, including transcription, epigenetics, and post-transcriptionally. By altering the EMT and hormone receptor pathways, changes in the expression of these lncRNAs have been linked to tumor growth, metastasis, and treatment resistance (82).

Similarly, by functioning as either tumor suppressors or oncogenes, miRNAs (microRNAs) aid in the regulation of breast cancer. Overexpression of miRNAs such as miR-21, miR-155, and miR-34a alters a cell’s capacity for self-destruction, pace of division, and immune system evasion. They are helpful in identifying changes in health because of their constant blood levels (83).

### 4.5 Highlighting the differences in detection technologies

IHC, NGS and ctDNA are different techniques that differ in both their technical features and how they are used in medicine see in Table 2. Using IHC, it’s easy to detect proteins in tissue samples and the process is not very costly, but detecting mutations is not straightforward. When compared to PGS, NGS is more sensitive and specific and provides a complete genomic profile, including mutations, extra and missing chromosome copies and gene fusions. However, you need to use advanced technical equipment and have trained specialists. Liquid biopsies, using ctDNA, offer a way to monitor changes in cancer without invasive procedures. On the other hand, they might be less sensitive toward early cancers and this could also depend on the quality of the sample and the amount of tumor material released by the tumor. For this reason, it is important to use the right technology for the situation and now, more and more, combine different platforms to increase the accuracy of diagnoses and treatment plans specific to each person.

**TABLE 2:** Comprehensive table summarizing detection technologies (e.g., IHC vs. NGS vs. ctDNA platforms) from the past 15 years.

Detection technology	Sensitivity	Specificity	Clinical applicability	Limitations	References
Immunohistochemistry (IHC)	Moderate (∼70–85%)	High (∼85–95%)	Detects protein expression in formalin-fixed tissue samples	Semi-quantitative; antibody-dependent; cannot detect gene mutations	(84)
Next-generation sequencing (NGS)	Very high (>95%)	Very high (>95%)	Comprehensive genomic profiling; detects mutations, fusions, and copy number variations	High cost; longer turnaround time; requires bioinformatics expertise	(7)
Circulating tumor DNA (ctDNA)	Variable (low to high; 60%–90%)	Moderate to high (∼80–95%)	Non-invasive liquid biopsy for disease monitoring and mutation detection	Low ctDNA levels in early disease; analytical variability; requires sensitive assays	(84)

## 5 Challenges in biomarker development

The issues that biomarker development encounters include low reliability across clinical centres, biomarker validation costs, flexibility borne by patients by genetic and lifestyle differences. Moreover, their translation from research to clinical practices involve no definite protocols and associated regulatory barriers hampering the incorporation of biomarkers into practice (85).

### 5.1 Tumors heterogeneity

Biomarkers can be unreliable when tumors are diverse both within themselves and across them and this is not examined enough in precision oncology. There are often multiple genetically separate groups of cells existing together within one tumor which can make it hard to define reliable and consistent markers. Since biomarkers vary, the information they provide may not cover all parts of the tumor process and may lower accuracy in detection and treatment. Also, differences in the tumor between primary and metastatic sites make it harder to implement biomarkers in clinical practice. This difference proves that one biomarker approach cannot be used for all types of cancer. As a result, precision oncology needs to use many approaches and adjust treatments to the individual, using multi-regional sampling, an integrated study of many types of molecules and continuous monitoring to ensure the accuracy of biomarkers. Though they are mentioned in the literature, these issues should be discussed further and new methods developed to get the most from biomarkers in personalized cancer treatment (6).

### 5.2 Lack of standardization

A major downside of biomarkers is that, mainly due to historical reasons, most biomarkers can be measured by using a range of techniques, or in many cases only one single technique, which hampers clinical applicability and reproducibility. Variation originated from sample collection, sample processing, and analysis platform that result in poor reproducibility of biomarker data across different laboratories/studies. This situation of shift makes biomarkers non-reproducible and non-validated very much antithetical to its usefulness in diagnostic, prognostic and therapeutic settings (86). Furthermore, a lack of standardization enhances the problem of implementing biomarkers into mainstream clinical practice, thereby slowing the development of personalized medicine. These have implications for standardising guidelines and harmonising methodologies for biomarker validation and for increasing the reliability and clinical practice applicability of biomarker-based strategies (87).

### 5.3 Cost and accessibility

The high costs of performing most biomarker tests and their general availability concern affect their implementation in everyday clinical practice. Such tests frequently involve using advanced technologies and the use of equipment that is often costly, and as such, the tests are impractical for numerous hospitals or clinics, particularly in the developing world (88). Thirdly, training a sufficient number of personnel and creating necessary facilities also escalates expenses and keeps the services scarce. Such barriers include the following, which lead to unfair distribution of precision medicine for the underserved populations (89). Cutting biomarker costs through innovation in technology, optimizing testing procedures, and increasing the diagnostic centers are some major strategies needed to ensure equal utilization of biomarker based approaches among more patients all over the world (90).

Biomarker testing-related issues with global health equality can be resolved by offering low-cost technology, applying the same techniques worldwide, promoting cost-effective testing, aiding initiatives in low-income nations, and cooperating to create training and policies that are friendly. The majority of research on modern biomarkers is conducted in wealthy countries, leaving middle- and low-income countries with limited resources searching for flexible and affordable diagnostic alternatives.

## 6 Conclusion and future directions

**FIGURE 5:**
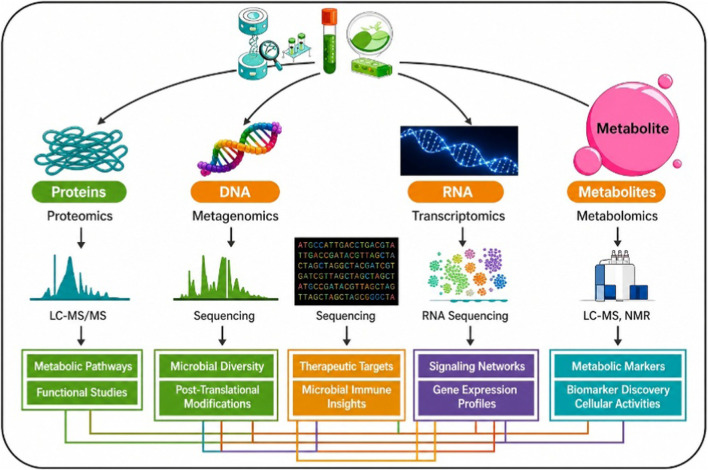
Multi-omics integration for advanced biomarker discovery and therapeutic insights.

According to this review, biomarkers are playing an increasingly important role in cancer detection, prognosis, and treatment selection. Although there are certain concerns with their widespread use, liquid biopsy and next-generation sequencing have the potential to significantly enhance customized cancer treatment. Next-generation sequencing could vastly improve tailored cancer treatment. While biomarkers may help with early detection and improved personalized treatment approaches, issues lie in the cost of testing, unreliability, and differing standards across the sector. The fusion of multi-omics strategies (proteomics, metagenomics, transcriptomics and metabolomics) and high-performance sequencing and mass spectrometry will allow a better understanding of molecular pathways and therapeutic targets, and precise biomarker discovery as depicted in Figure 5.

Remember, though: There is still much more research to be done in all such groups, with more people and other kinds of treatments being used and biomarkers matching ones elsewhere. Biomarker use in clinical practice remains difficult to exploit and requires enhancement.

Consequently, biomarkers have great potential to assist future oncology research, but the realization of this promise will be dependent on technology, regulatory approval, and cost efficiency. In conclusion, biomarkers have great potential for treating cancer, but broad application in medicine will take time and efforts. By harnessing novel technologies and analytical techniques in cancer biomarker studies, we anticipate substantial progress in the future. Specifically, in this direction of investigation the use of AI–based biomarker discovery in the quest to analyze and interpret large set of biomedical datasets efficiently is anticipated. Furthermore, combining approaches of multi-omics--genomics, transcriptomics, proteomics and metabolomics and a closer look at such networked interactions in cancer biology and biomarker biology, may offer us greater understanding. Next-generation technologies such as spatial transcriptomics will also allow researchers to examine gene expression over the space of tumor microenvironments. In addition, the growing use of liquid biopsy and next-generation sequencing (NGS) will help in early diagnosis, individualized diagnosis, accurate prognosis, and targeted therapy. These technologies are anticipated to become pivotal in advancing cancer diagnosis, drug selection, therapeutic decision making and overall patient prognosis as they are advancing and developed in more accessible manner.
